# Anti-Inflammatory Activities of Inotilone from *Phellinus linteus* through the Inhibition of MMP-9, NF-κB, and MAPK Activation *In Vitro* and *In Vivo*

**DOI:** 10.1371/journal.pone.0035922

**Published:** 2012-05-08

**Authors:** Guan-Jhong Huang, Shyh-Shyun Huang, Jeng-Shyan Deng

**Affiliations:** 1 School of Chinese Pharmaceutical Sciences and Chinese Medicine Resources, College of Pharmacy, China Medical University, Taichung, Taiwan; 2 Department of Pharmacy, College of Pharmacy, China Medical University, Taichung, Taiwan; 3 Department of Health and Nutrition Biotechnology, Asia University, Taichung, Taiwan; National Institutes of Health, United States of America

## Abstract

Inotilone was isolated from *Phellinus linteus.* The anti-inflammatory effects of inotilone were studied by using lipopolysaccharide (LPS)-stimulated mouse macrophage RAW264.7 cells and λ-carrageenan (Carr)-induced hind mouse paw edema model. Inotilone was tested for its ability to reduce nitric oxide (NO) production, and the inducible nitric oxide synthase (iNOS) expression. Inotilone was tested in the inhibitor of mitogen-activated protein kinase (MAPK) [extracellular signal-regulated protein kinase (ERK), c-Jun NH_2_-terminal kinase (JNK), p38], and nuclear factor-κB (NF-κB), matrix-metalloproteinase (MMP)-9 protein expressions in LPS-stimulated RAW264.7 cells. When RAW264.7 macrophages were treated with inotilone together with LPS, a significant concentration-dependent inhibition of NO production was detected. Western blotting revealed that inotilone blocked the protein expression of iNOS, NF-κB, and MMP-9 in LPS-stimulated RAW264.7 macrophages, significantly. Inotilone also inhibited LPS-induced ERK, JNK, and p38 phosphorylation. In *in vivo* tests, inotilone decreased the paw edema at the 4^th^ and the 5^th^ h after Carr administration, and it increased the activities of catalase (CAT), superoxide dismutase (SOD), and glutathione peroxidase (GPx). We also demonstrated that inotilone significantly attenuated the malondialdehyde (MDA) level in the edema paw at the 5^th^ h after Carr injection. Inotilone decreased the NO and tumor necrosis factor (TNF-α) levels on serum at the 5^th^ h after Carr injection. Western blotting revealed that inotilone decreased Carr-induced iNOS, cyclooxygenase-2 (COX-2), NF-κB, and MMP-9 expressions at the 5^th^ h in the edema paw. An intraperitoneal (*i.p.*) injection treatment with inotilone diminished neutrophil infiltration into sites of inflammation, as did indomethacin (Indo). The anti-inflammatory activities of inotilone might be related to decrease the levels of MDA, iNOS, COX-2, NF-κB, and MMP-9 and increase the activities of CAT, SOD, and GPx in the paw edema through the suppression of TNF-α and NO. This study presents the potential utilization of inotilone, as a lead for the development of anti-inflammatory drugs.

## Introduction

Inflammation, a physiological response to infection or injury, plays a critical role in chronic diseases, including asthma, rheumatoid arthritis, atherosclerosis, and Alzheimer’s disease, and it plays a role in various human cancers [1]. Among its mediators, inducible nitric oxide synthase (iNOS) and cyclooxygenase-2 (COX-2) are important enzymes that regulate inflammatory processes [2]. In addition, one of the major factors involved in the inflammation response is induced by lipopolysaccharide (LPS) and various inflammatory mediator cytokines such as interferon, interleukins, and tumor necrosis factor (TNF)-α [3]. Many researchers reported that inflammatory effect induced by λ-carrageenan (Carr) could be associated with free radical formation. Free radical, prostaglandin and NO will be released when administrating with Carr for 1–5 h. The edema effect was raised to maximum at the 3^th^ h and its malondialdehyde (MDA) production was due to free radical attack plasma membrane [4]. Therefore, in this paper, we examined the anti-inflammatory effects of inotilone on LPS-induced RAW264.7 cells and Carr-induced paw edema in mice.

The generation of reactive oxygen species (ROS) has been shown to modulate both the expression and activity of MMPs [5]. ROS also increases the expression of MMPs via cell signaling pathways, such as the mitogen activated protein kinase (MAPK) pathways that are regulated by redox-sensitive phosphatases [6]. MAPK pathways are the evolutionarily conserved kinase module that links extracellular signals to the machinery controlling fundamental cellular processes such as growth, proliferation, differentiation, and cell death [7]. An important amount of evidence has indicated that macrophages under certain stimuli induce matrix metalloproteinase 9 (MMP-9) expression and protein secretion through the activation of extracellular signal-regulated protein kinase (ERK) and nuclear factor-κB (NF-κB) signaling pathways [5]. MMP-9 expression in macrophages, mediates cell migration and proliferation by promoting extracellular matrix remodeling [8].

*Phellinus linteus* (Berk. & M.A. Curt.) (PL) is a mushroom that belongs to the genus *Phellinus* and it is commonly called “Sangwhang” in Taiwan. It is popular in oriental countries and has been traditionally used as food and medicine. PL contains many bioactive compounds, and it is known to prevent various diseases, such as cancer, ulcer, bacterial and viral infections and diabetes [9]. Recently, PL has been exhibited various biological activities, including anti-oxidative, anti-inammatory, cytotoxic, anti-platelet aggregation, anti-diabetic, anti-dementia, and anti-viral effects [10]. PL has been isolated several aromatic compounds from the cultured mycelia such as hydroxybenzaldehyde, caffeic acid, hispolon, hispidin, and inotilone [11]. We recently reported that inotilone had previously been shown to possess anti-inflammatory [12], and α-glucosidase and aldose reductase inhibitory activities [9]. This study examined the anti-inflammatory effects of inotilone by using LPS-stimulated RAW264.7 cell *in vitro* and Carr-induced mouse paw edema model *in vivo*. The study also evaluated the effect of inotilone on MMP-9 expression associated NF-κB and MAPK signaling pathways to reveal molecular mechanism.

## Methods

### Chemicals

LPS (endotoxin from *Escherichia coli*, serotype 0127:B8), Carr (Type IV), Indo, MTT (3-[4, 5-dimethylthiazol-2-yl]-2, 5-diphenyltetrazolium bromide) and other chemicals were purchased from Sigma Chemical Co. (St. Louis, USA). TNF-α was purchased from Biosource International Inc. (Camarillo, CA, USA). Anti-iNOS, anti-COX-2, anti-β-actin antibody (Santa Cruz, USA) and a protein assay kit (Bio-Rad Laboratories Ltd., Watford, Herts, U.K.) were obtained as indicated. Poly-(vinylidene fluoride) membrane (Immobilon-P) was obtained from Millipore Corp. (Bedford, MA, USA). The antibody against MMP-9, NF-κB, ERK, JNK, and p38 proteins and phosphorylated proteins were purchased from Cell Signaling Technology (Beverly, MA).

### Isolation and Characterization of Inotilone from Fruiting Body of PL

The fruiting body of PL (about 1.0 kg, air dry weight) was powdered, and extracted with 6 L 95% EtOH at room temperature (3 times, 72 h each). Extracts were filtered and combined together, and then evaporated at 40°C (N-11, Eyela, Japan) to dryness under reduced pressure to give a dark brown residue (40 g). The yield obtained for PL is about 4%. The crude extract was suspended in H_2_O (1 L), and then partitioned with 1 L *n*-hexane (×2), 1 L EtOAc (×2) and 1 L *n*-butanol (×2), successively.

Inotilone was purified from the EtOAc soluble portion (8 g) by a bioassay-guid separation. A portion of the active EtOAc fraction was subjected to silica gel chromatography using stepwise CHCl_3_-MeOH (9∶1, 8∶2, 1∶1 *v/v*) as eluent. Final purification was achieved by preparative HPLC (Spherisorb ODS-2 RP18, 5 µm (Promochem), 250×25 mm, acetonitrie-H_2_O (83∶17 *v/v*), at a flow rate of 10 mL/min and UV detection at 375 nm). The identification of inotilone was performed by comparing their physical spectral data with literature values [9].

#### Inotilone

^1^H NMR (400 MHz, DMSO) δ 2.55 (s, 3 H, CH_3_), 5.80 (s, 1 H, CH), 6.49 (s, 1 H, CH), 6.80 (d, 1 H, *J* = 8.4 Hz, ArH), 7.16 (dd, 1 H, *J* = 8.4, 2.0 Hz, ArH), 7.34 (d, 1 H, *J* = 2.0 Hz, ArH); ^13^C NMR (100 MHz, DMSO) δ 15.9, 105.7, 112.3, 116.2, 118.2, 123.1, 125.0, 144.6, 145.7, 148.4, 180.9, 187.0.

### Animals

Imprinting control region (ICR; 6–8 weeks male) mice were obtained from the BioLASCO Taiwan Co., Ltd. The animals were kept in plexiglass cages at a constant temperature of 22±1°C, and relative humidity of 55±5% with 12 h dark-light cycle for at least 2 weeks before the experiment. They were given food and water *ad libitum*. This animal study was approved by the Institutional Animal Care and Use Committee (IACUC) of the China Medical University, Taiwan, and all animal procedures were performed according to the IACUC policy. And the recommendations of the Committee for Research and Ethical Issues of the International Association for the Study of Pain (IASP) Ethical Guidelines (Committee for Research and Ethical Issues of the IASP, 1983) were adhered in these studies [13]. In particular, the duration of the experiments was as short as possible and the number of animals was kept to a minimum.

After a 2-week adaptation period, male ICR mice (18–25 g) were randomly assigned to four groups (n = 6) of the animals in the study. The control group received normal saline (intraperitoneal; *i.p.*). The other three groups included a Carr-treated, a positive control (Carr + Indo) and inotilone administered groups (Carr + inotilone).

### Cell Culture

A murine macrophage cell line RAW264.7 (BCRC No. 60001) was purchased from the Bioresources Collection and Research Center (BCRC) of the Food Industry Research and Development Institute (Hsinchu, Taiwan). Cells were cultured in plastic dishes containing Dulbecco’s Modified Eagle Medium (DMEM, Sigma, St. Louis, MO, USA) supplemented with 10% fetal bovine serum (FBS, Sigma, USA) in a CO_2_ incubator (5% CO_2_ in air) at 37°C and subcultured every 3 days at a dilution of 1∶5 using 0.05% trypsin–0.02% EDTA in Ca^2+^-, Mg^2+^- free phosphate-buffered saline (DPBS).

### Cell Viability

Cells (2×10^5^) were cultured in 96-well plate containing DMEM supplemented with 10% FBS for 1 day to become nearly confluent. Then cells were cultured with inotilone in the presence of 100 ng/mL LPS for 24 h or 1 h. After that, the cells were washed twice with DPBS and incubated with 100 µL of 0.5 mg/mL MTT for 2 h at 37°C testing for cell viability. The medium was then discarded and 100 µL dimethyl sulfoxide (DMSO) was added. After 30-min incubation, absorbance at 570 nm was read by using a microplate reader (Molecular Devices, Orleans Drive, Sunnyvale, CA).

### Measurement of Nitric Oxide/Nitrite

NO production was indirectly assessed by measuring the nitrite levels in the cultured media and serum determined by a colorimetric method based on the Griess reaction [14]. The cells were incubated with inotilone (0, 1.56, 3.12, 6.25, 12.5, and 25 µM) in the presence of LPS (100 ng/mL) at 37°C for 24 h. Then, cells were dispensed into 96-well plates, and 100 µL of each supernatant was mixed with the same volume of Griess reagent (1% sulfanilamide, 0.1% naphthyl ethylenediamine dihydrochloride and 5% phosphoric acid) and incubated at room temperature for 10 min, the absorbance was measured at 540 nm with a Micro-Reader (Molecular Devices, Orleans Drive, Sunnyvale, CA). Serum samples were diluted four times with distilled water and deproteinized by adding 1/20 volume of zinc sulfate (300 g/L) to a final concentration of 15 g/L. After centrifugation at 10,000×*g* for 5 min at room temperature, 100 µL supernatant was applied to a microtiter plate well, followed by 100 µL of Griess reagent. After 10 min of color development at room temperature, the absorbance was measured at 540 nm with a Micro-Reader. By using sodium nitrite to generate a standard curve, the concentration of nitrite was measured form absorbance at 540 nm.

### Determination of MMP-9 by Zymography

MMP in the medium released from RAW264.7 cells was assayed using gelatin zymography (7.5% zymogram gelatin gels) according to the methods reported by Liao et al. (2006) [15] with some modification. Briefly, the culture medium was electrophoresed (120 V for 90 min) in a 10% SDS-PAGE gel containing 0.1% gelatin. The gel was then washed at room temperature in a solution containing 2.5% (*v/v*) Triton X-100 with two changes and subsequently transferred to a reaction buffer for enzymatic reaction containing 1% NaN_3_, 10 mM CaCl_2_ and 40 mM Tris–HCl, pH 8.0, at 37°C with shaking overnight (for 12–15 h). Finally, the MMP gel was stained for 30 min with 0.25% (*w/v*) Coomassie blue in 10% acetic acid (*v/v*) and 20% methanol (*v/v*) and destained in 10% acetic acid (*v/v*) and 20% methanol (*v/v*).

### Carr-induced Edema

The Carr-induced hind paw edema model was used for determination of anti-inflammatory activity [16]. Animals were *i.p.* treated with inotilone (1.25, 2.50, and 5 mg/kg), Indo (10 mg/kg) or normal saline, 30 min prior to injection of 1% Carr (50 µL) in the plantar side of right hind paws of the mice. The paw volume was measured immediately after Carr injection and at 1, 2, 3, 4, and 5 h intervals after the administration of the edematogenic agent using a plethysmometer (model 7159, Ugo Basile, Varese, Italy). The degree of swelling induced was evaluated by the ratio a/b, where was the volume of the right hind paw after Carr treatment, and b was the volume of the right hind paw before Carr treatment. Indo was used as a positive control. After 5 hrs, the animals were sacrificed and the Carr-induced edema feet were dissected and stored at −80°C. Also, blood were withdrawn and kept at −80°C.

In the secondary experiment, the right hind paw tissue and paw edema tissue took at the 5^th^ h. The right hind paw tissue was rinsed in ice-cold normal saline, and immediately placed in cold normal saline four times their volume and homogenized at 4°C. Then the homogenate was centrifuged at 12,000×*g* for 5 min. The supernatant was obtained and stored at −20°C for MDA assays. The whole paw edema tissue was rinsed in ice-cold normal saline, and immediately placed in cold normal saline one time their volume and homogenized at −4°C. Then the homogenate was centrifuged at 12,000×*g* for 5 min. The supernatant was obtained and stored at −20°C for the antioxidant enzymes (CAT, SOD and GPx) activity assays. The protein concentration of the sample was determined by the Bradford dye-binding assay (Bio-Rad, Hercules, CA).

### MDA Assay

MDA from Carr-induced edema foot was evaluated by the thiobarbituric acid reacting substances (TBARS) method [17]. Briefly, MDA reacted with thiobarbituric acid in the acidic high temperature and formed a red-complex TBARS. The absorbance of TBARS was determined at 532 nm.

### Measurement of Serum TNF-α by an Enzyme-Linked Immunosorbent Assay (ELISA)

Serum levels of TNF-α were determined using a commercially available ELISA kit (Biosource International Inc., Camarillo, CA) according to the manufacturer’s instruction. TNF-α was determined from a standard curve. The concentrations were expressed as pg/mL.

### Antioxidant Enzyme Activity Measurements

The following biochemical parameters were analyzed to check the antioxidant enzyme activity of inotilone in the paw edema by the methods given below.

Total SOD activity was determined by the inhibition of cytochrome *c* reduction [18]. The reduction of cytochrome *c* was mediated by superoxide anions generated by the xanthine/xanthine oxidase system and monitored at 550 nm. One unit of SOD was defined as the amount of enzyme required to inhibit the rate of cytochrome *c* reduction by 50%. Total CAT activity was based on that of Aebi [19]. In brief, the reduction of 10 mM H_2_O_2_ in 20 mM of phosphate buffer (pH 7) was monitored by measuring the absorbance at 240 nm. The activity was calculated using a molar absorption coefficient, and the enzyme activity was defined as nanomoles of dissipating hydrogen peroxide per milligram protein per minute. Total GPx activity in cytosol was determined according to Paglia and Valentine’s method [20]. The enzyme solution was added to a mixture containing hydrogen peroxide and glutathione in 0.1 mM Tris buffer (pH 7.2) and the absorbance at 340 nm was measured. The activity was calculated by using a calibration curve of GPx established from bovine whole blood. A linear relationship between the activity (unit/mL) of GPx and the reduction of NADPH absorbance at 340 nm was found and the enzyme activity was defined as nanomoles of NADPH oxidized per milligram protein per minute.

### Protein Lysate Preparation and Western Blot Analysis

The stimulated murine macrophage cell line RAW264.7 cells were washed with PBS and lysed in an ice-cold lysis buffer [10% glycerol, 1% Triton X-100, 1 mM Na_3_VO_4_, 1 mM EGTA, 10 mM NaF, 1 mM Na_4_P_2_O_7_, 20 mM Tris buffer (pH 7.9), 100 mM β-glycerophosphate, 137 mM NaCl, 5 mM EDTA, and one protease inhibitor cocktail tablet (Roche, Indianapolis, IN, USA)] on ice for 1 h, followed by centrifugation at 12,000×*g* for 30 min at 4°C. Soft tissues were removed from individual mice paws and homogenized in a solution containing 10 mM CHAPS, 1 mM phenylmethylsulphonyl fluoride (PMSF), 5 µg/mL, aprotinin, 1 µM pepstatin and 10 µM leupeptin. The homogenates were centrifuged at 12,000×*g* for 20 min, and 30 µg of protein from the supernatants was then separated on 10% sodium dodecylsulphate–polyacrylamide gel (SDS-PAGE) and transferred to polyvinylidene difluoride membranes. After transfer, the membrane was blocked for 2 h at room temperature with 5% skim milk in Tris-buffered saline-Tween (TBST; 20 mM Tris, 500 mM NaCl, pH 7.5, 0.1% Tween 20). The membranes were then incubated with antibody in 5% skim milk in TBST for 2 h at room temperature. The membranes were washed three times with TBST at room temperature and then incubated with a 1∶2000 dilution of anti-mouse IgG secondary antibody conjugated to horseradish peroxidase (Sigma, St Louis, MO, U.S.A.) in 2.5% skim milk in TBST for 1 h at room temperature. The membranes were washed three times and the immunoreactive proteins were detected by enhanced chemiluminescence (ECL) by using hyperfilm and ECL reagent (Amersham International plc., Buckinghamshire, U.K.). The results of Western blot analysis were quantified by measuring the relative intensity compared to the control using Kodak Molecular Imaging Software (Version 4.0.5, Eastman Kodak Company, Rochester, NY) and represented in the relative intensities.

### Histological Examination

For histological examination, biopsies of paws took 5 hrs following the intraplantar injection of Carr. The tissue slices were fixed in Dietric solution (14.25% ethanol, 1.85% formaldehyde, 1% acetic acid) for 1 week at room temperature, dehydrated by graded ethanol and embedded in Paraplast (Sherwood Medical). Sections (7 µm thick) were deparaffinized with xylene and stained with trichromic Van Gieson, and antigen retrieval was performed with citrate buffer, then blocked with 5% normal goat serum in PBS and incubated with rabbit anti-COX-2 and anti-iNOS in PBS with 5% normal goat serum. The sections were incubated with biotinylated goat anti-rabbit IgG. After washing in PBS, sections were processed with the Dako kit (Dako REALTM envision TM detection system). Thus, some sections were stained with hematoxylin and eosin, while others were processed for iNOS and COX-2 immunohistochmistry staining. All samples were observed and photographed with BH2 Olympus microscopy. Every three to five tissue slices were randomly chosen from Control, Carr, Indo and inotilone-treated (5 mg/kg) groups [21].

### Statistical Analysis

Experimental results were presented as the mean ± standard deviation (SD) of three parallel measurements. IC_50_ values were estimated using a non-linear regression algorithm (SigmaPlot 8.0; SPSS Inc. Chicago, IL). Data obtained from animal experiments were expressed as mean standard error (±S.E.M.). Statistical evaluation was carried out by one-way analysis of variance (ANOVA followed by Scheffe’s multiple range tests). Statistical significance is expressed as ^*^ *p*<0.05, ^**^ *p*<0.01 and^ ***^ *p*<0.001.

## Results

### Isolation of Inotilone from PL and its Structural Characterization

**Figure 1:**
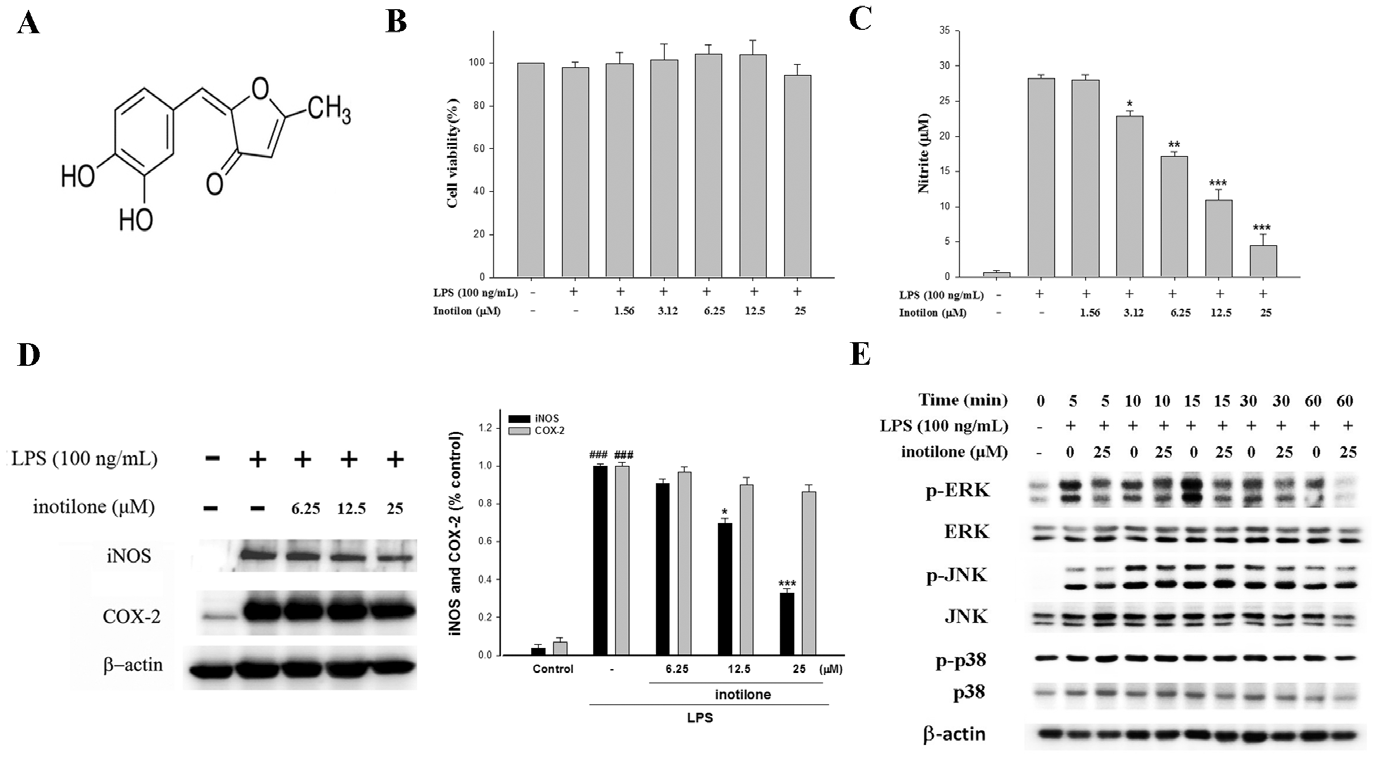
The chemical structure of inotilone (A) and the effects of inotilone on lipopolysaccharide (LPS)-induced cell viability (B), NO production (C), inhibition of iNOS and COX-2 protein expression (D), and MAPK (JNK, p38, and ERK) protein expression (E) were evaluated in RAW264.7 cells. Cells were incubated for 24 h or 5, 10, 15, 30, and 60 mins with 100 ng/mL of LPS in the absence or presence of inotilone (0, 1.56, 3.12, 6.25, 12.5, and 25 µM). Inotilone was added 1 h before the incubation with LPS. Cell viability was performed by using MTT assay. Nitrite concentration in the medium was determined by using Griess reagent. Lysed cells were then prepared and subjected to Western blotting by using an antibody specific for iNOS, COX-2, and MAPK. β-actin was used as an internal control. The data were presented as mean ± S.D. for three different experiments performed in triplicate. **p<0.01 and ***p<0.001 were compared with LPS-alone group.

PL was isolated via extensive chromatographic purification of the ethyl acetate-soluble fraction of the dried fruiting body. The chemical structure of the purified yellow powder was elucidated by NMR spectroscopy and mass spectrometry studies and it was identified as inotilone (Fig. 1A) [9].

### Cell Viability and Effect of Inotilone on LPS-induced NO Production in Macrophages

The effect of inotilone on RAW264.7 cell viability was determined by a MTT assay. Cells cultured with inotilone at the concentrations (0, 1.56, 3.12, 6.25, 12.5, and 25 µM) used in the presence of 100 ng/mL LPS for 24 h did not change cell viability (Fig. 1B). Inotilone did not interfere with the reaction between nitrite and Griess reagents at 25 µM (data not shown). Unstimulated macrophages, after 24 h of incubation in culture medium produced background levels of nitrite. When RAW264.7 macrophages were treated with different concentrations of inotilone (0, 1.56, 3.12, 6.25, 12.5, and 25 µM) together with LPS (100 ng/mL) for 24 h, a significant concentration-dependent inhibition of nitrite production was detected. There was either a significant decrease in the nitrite production of group treated with 3.12 µM inotilone (*p*<0.05) or very or highly significant decrease of groups treated respectively with 6.25, 12.5 and 25 µM of inotilone when compared with the LPS-alone group (*p*<0.01 or *p*<0.001). The IC_50_ value for inhibition of nitrite production of inotilone was about 10.24±0.35 µM (Fig. 1C).

### Inhibition of LPS-induced iNOS and COX-2 Protein by Inotilone

In order to investigate whether the inhibition of NO production was due to a decreased iNOS and COX-2 protein level, the effect of inotilone on iNOS and COX-2 protein expression was studied by immunoblot. The results showed that incubation with inotilone (0, 6.25, 12.5, and 25 µM) in the presence of LPS (100 ng/mL) for 24 hrs inhibited iNOS proteins expression in mouse macrophage RAW264.7 cells in a dose-dependent manner (Fig. 1D). The detection of β-actin was also performed in the same blot as an internal control. The intensity of protein bands were analyzed using Kodak Quantity software in three independent experiments and showed an average of 67.1 and 13.6% down-regulation of iNOS and COX-2 proteins after treatment with inotilone at 25µM compared with the LPS-alone.

### Effects of Inotilone on the LPS-stimulated Activation of Mitogen-activated Protein Kinases (MAPKs)

MAPKs play critical roles in the regulation of cell growth and differentiation, and control cellular responses to cytokines and stresses. In particular, ERK, p38, and JNK are known to be important for the activation of NF-κB [21], [22]. To explore whether the inhibition of NF-κB activation by inotilone is mediated through the MAPK pathway, MAPK phosphorylation was examined by Western blot in RAW 264.7 cells pretreated with inotilone and then with LPS. As shown in Fig. 1E, inotilone suppressed the LPS-induced activation of ERK, JNK, and p38 MAPKs in a time-dependent manner. However, the expression of non-phosphorylated ERK, JNK, and P38 MAPKs was unaffected by LPS or LPS plus inotilone. These results suggest that phosphorylation of MAPKs may be involved in the inhibitory effect of inotilone on LPS-stimulated NF-κB binding in RAW 264.7 cells.

### Inhibition of LPS-induced MMP and NF-κB Proteins by Inotilone

**Figure 2:**
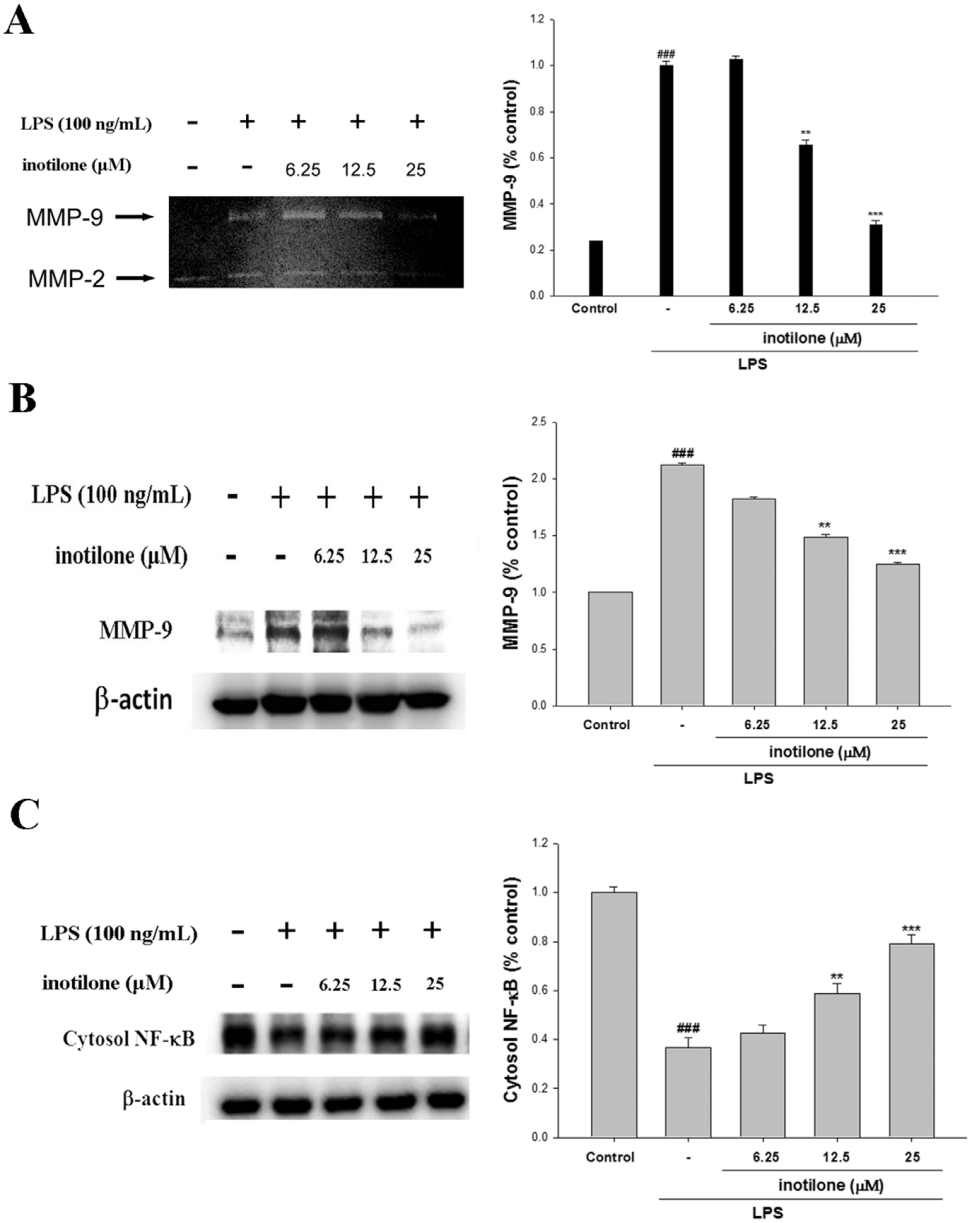
Inotilone suppresses LPS-induced MMP-9 activities (A), MMP-9 protein (B), and NF-κB expressions (C) in RAW264.7 cells. Cells were incubated for 24 h or 1 h with 100 ng/mL of LPS in the absence or the presence of inotilone (0, 6.25, 12.5, and 25 µM). Inotilone was added 1 h before the incubation with LPS. The conditioned media were collected MMP-9 activities determined by gelatin zymography. MMP-9 activities were quantified by densitometric analysis. Representative Western blot from two separate experiments was shown. MMP-9 and NF-κB levels were calculated with reference to a LPS-stimulated culture. The data were presented as mean ± S.D. for three different experiments performed in triplicate.^ ###^compared with sample of control group. **p<0.01 and ***p<0.001 were compared with LPS-alone group.

The effect of inotilone on MMP-9 activation was analyzed by gelatin zymography and immunoblot. As shown in Fig. 2A, the results showed that the incubation with inotilone (0, 6.25, 12.5, and 25 µM) in the presence of LPS for 24 h MMP-9 activation in mouse macrophage RAW264.7 cells in a dose-dependent manner. The intensity of protein bands were analyzed by using Kodak Quantity software in three independent experiments and showed an average of 68.1% down-regulation of MMP-9 activation after the treatment with inotilone at 25µM compared with the LPS-alone. The effect of MMP-9 expression by inotilone in the presence of LPS (100 ng/mL) for 24 h was assessed by Western blotting. The results showed the incubation with inotilone (0, 6.25, 12.5, and 25 µM) in the presence of LPS for 24 h inhibited MMP-9 proteins expression in mouse macrophage RAW264.7 cells in a dose-dependent manner (Fig. 2B). The detection of β-actin was also performed in the same blot as an internal control. The intensity of protein bands was analyzed by using Kodak Quantity software in three independent experiments and the result showed an average of 58.9% down-regulation of MMP-9 proteins, respectively, after the treatment with inotilone at 25 µM compared with the LPS-alone.

The effect of NF-κB expression by inotilone in the presence of LPS for 1 h was assessed by Western blotting. And the intensity of protein bands showed an average of 79.2% increase of NF-κBprotein after treatment with inotilone at 25 µM compared with the LPS-alone (Fig. 2C). Therefore, it can be concluded that inotilone is capable of inhibiting iNOS expression in LPS induced RAW264.7 cells via attenuation of NF-κB signaling by ERK, p38, and JNK.

### Effects of Inotilone on Carr-induced Mouse Paw Edema

**Figure 3:**
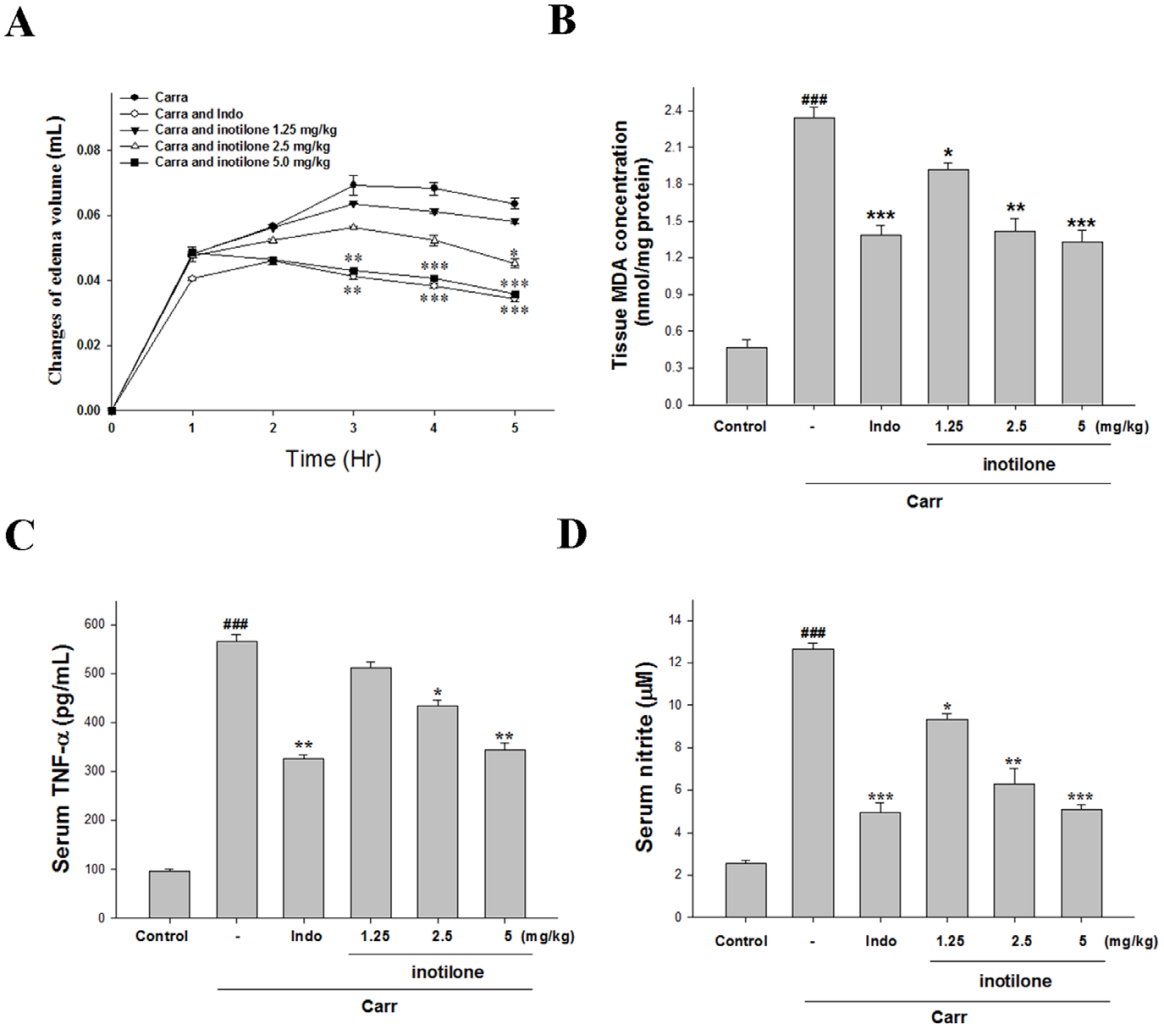
Effects of inotilone and Indo on hind paw edema induced by Carr in mice (A), the tissue MDA concentration of foot in mice (B), Carr-induced NO (C), and TNF-α (D) concentrations of serum at the 5^th^ **h in mice.** Each value represents as mean ± S.E.M. ^###^ *p*<0.001 as compared with the control group. *p<0.05, **p<0.01, and ***p<0.001 as compared with the Carr group (one-way ANOVA followed by Scheffe’s multiple range test).

Because inotilone effectively inhibited iNOS inductions in macrophages, studies were extended to determine whether inotilone affected acute phase inflammation in animal models. In this study, we used Carr-induced edema because this model is widely employed for screening the effects of anti-inflammatory drugs. Carr-induced paw edema is shown in Fig. 3A. Inotilone (5 mg/kg) inhibited (*p*<0.001) the development of paw edema induced by Carr (10 mg/kg) at the 4^th^ h and the 5^th^ h after the treatment, significantly. Inotilone at the concentration of 5 mg/kg, the levels of edema volume were decreased to 56.2% of that observed in the Carr alone group. Indo (10 mg/kg) significantly decreased the Carr induced paw edema at the 4^th^ h and the 5^th^ h after the treatment (*p*<0.001).

### Effects of Inotilone on the MDA Level

The MDA level increased significantly in the edema paw at the 5^th^ h after Carr injection (*p*<0.001). However, the MDA level was decreased significantly by treatment with inotilone (5 mg/kg) (*p*<0.001), as well as 10 mg/kg Indo (Fig. 3B). The inhibition mice MDA levels compared with the Carr group are 18.3%, 39.7%, and 43.3%, respectively.

### Effects of Inotilone on the TNF-α Level

The TNF-*α* level increased significantly in serum at the 5^th^ h post-Carr injection (*p*<0.001). However, inotilone (5 mg/kg) decreased the TNF-α level in serum at the 5^th^ h after Carr injection (*p*<0.01), as well as 10 mg/kg Indo (Fig. 3C). In the range of 1.25–5 mg/kg, inotilone could inhibit the level of TNF-*α* to 10.6–40.3% of the observation in Carr group.

### Effects of Inotilone on the NO Level

In Fig. 3D, the NO level increased significantly in the edema serum at the 5^th^ h post-Carr injection (*p*<0.001). Inotilone (5 mg/kg) significantly decreased the serum NO level (*p*<0.001). Meanwhile, in the range of 1.25–5 mg/kg, inotilone could inhibit the level of nitrite to 26.2–59.7% of the observation in Carr group. The inhibitory potency was similar to that of Indo (10 mg/kg) at the 5^th^ h after induction.

### Effects of Inotilone on Activities of Antioxidant Enzymes

At the 5^th^ h after the intrapaw injection of Carr, paw edema tissues were also analyzed for the biochemical parameters such as CAT, SOD, and GPx activities. Carr decreased the activities of CAT, SOD, and GPx in paw edema by 29.3%, 33.9%, and 32.1%, respectively, in comparison to control group. In the range of 1.25–5 mg/kg, inotilone could increase the activities of CAT to 114.3%–125.6%, SOD to 108.2%–139.6%, and GPX to 108.8%–123.5%, respectively, of that observed in Carr along group. Indo also exhibited increase effects in the activities of CAT (129.2%), SOD (140.7%), and GPx (124.1%) in comparison to Carr group (*P*<0.01) (Table 1). These data implied that the protective effects of inotilone might be attributed to its elevation in the antioxidant enzymes activities of Carr induced mice.

**Table 1:** Effects of inotilone and indomethacin (Indo) on changes in CAT, SOD and GPx activities was studied on Carr-induced mice paw edema (5^th^ h).

Groups	Catalase (U/mg protein)	SOD (U/mg protein)	GPx (U/mg protein)
Control	4.75±0.31	25.28±0.23	21.96±0.28
Carr	3.36±0.23^###^	16.71±0.18^###^	14.92±0.13^###^
Carr + Indo	4.34±0.16**	23.51±0.14**	18.52±0.23**
Carr + inotilone (1.25 mg/Kg)	3.84±0.22	18.08±0.08	16.24±0.13
Carr + inotilone (2.5 mg/Kg)	4.07±0.18*	21.43±0.25*	17.85±0.37*
Carr + inotilone (5 mg/Kg)	4.22±0.25**	23.33±0.21**	18.43±0.24**

Each value represents as mean ± S.E.M.^ ###^ *p*<0.001 as compared with the control group. *p<0.05 and **p<0.01 as compared with the Carr group (one-way ANOVA followed by Scheffe’s multiple range test).

### Effects of Inotilone on Carr-induced iNOS and COX-2 Protein Expression in Mouse Paw Edema

**Figure 4:**
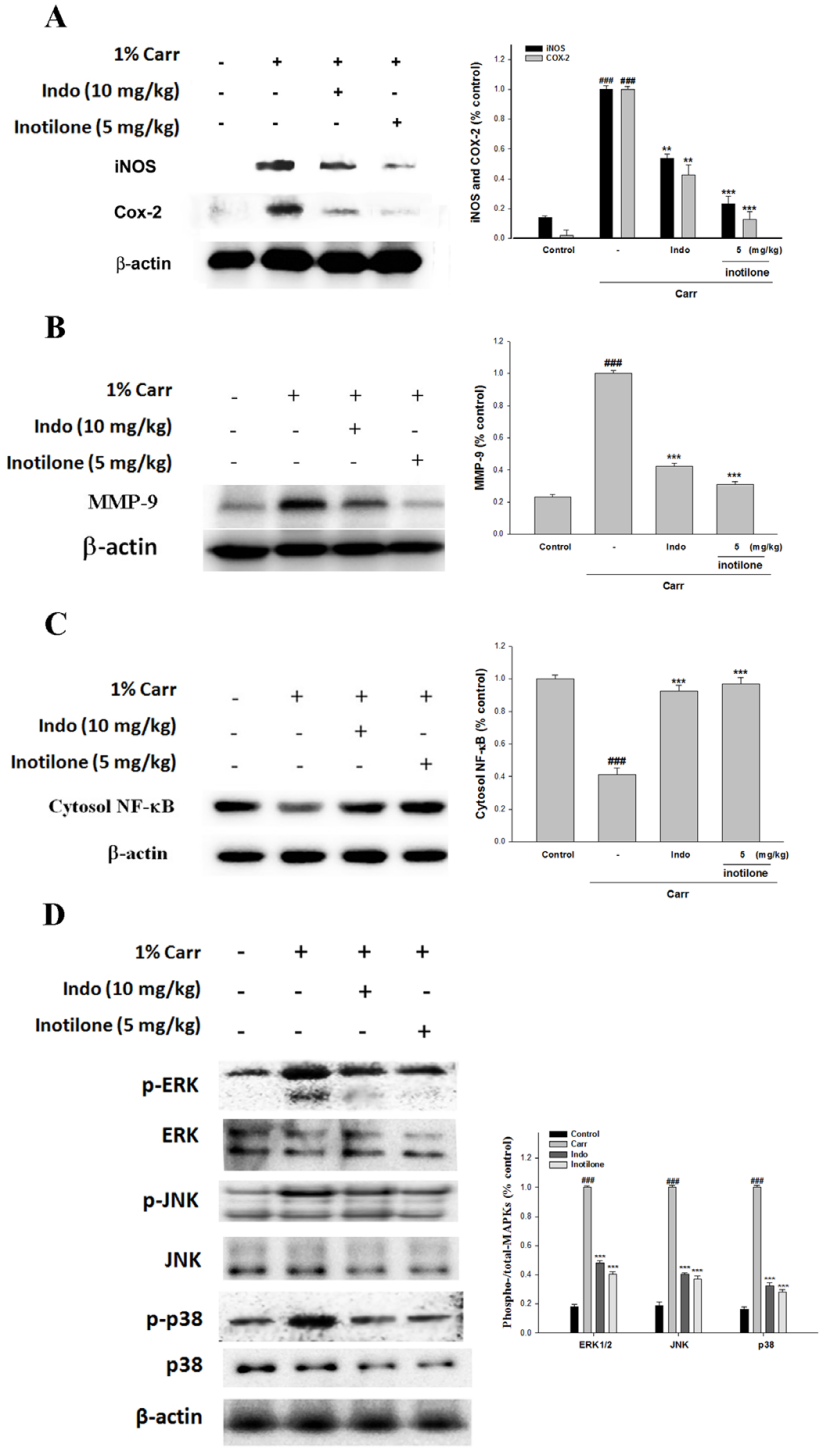
Inhibition of iNOS, COX-2 (A), MMP-9 (B), NF-κB (C), and MAPK (JNK, p38, and ERK) (D) protein expressions by inotilone induced by Carr of foot at the 5^th^ **h in mice.** Suspended tissue were then prepared and subjected to Western blotting using an antibody specific for iNOS and COX-2. β-actin was used as an internal control. A representative Western blot from two separate experiments is shown. Relative iNOS, COX-2, MMP-9, NF-κB, and MAPK (JNK, p38, and ERK) protein levels were calculated with reference to a Carr-injected mouse. The data were presented as mean ± S.D. for three different experiments performed in triplicate. ^###^ *p*<0.001 as compared with the control group. *p<0.05, **p<0.01 and ***p<0.001 as compared with the Carr group (one-way ANOVA followed by Scheffe’s multiple range test).

To investigate whether the inhibition of NO production was due to a decreased iNOS and COX-2 protein level, the effect of inotilone on iNOS and COX-2 proteins expression were studied by Western blot. The results showed that the injection of inotilone (5 mg/kg) on Carr-induced for 5 h inhibited iNOS and COX-2 proteins expression in mouse paw edema (Fig. 4A). The intensity of protein bands was analyzed by using Kodak Quantity software in three independent experiments and showed an average of 76.7% and 87.2% reduction of iNOS and COX-2 protein, respectively, after treatment with inotilone at 5 mg/kg compared with the Carr-induced alone. In addition, the protein expression showed an average of 46.1% and 57.3% reduction of iNOS and COX-2 protein after the treatment with Indo at 10.0 mg/kg compared with the Carr-induced alone. The down-regulation of iNOS and COX-2 activity of the inotilone (5 mg/kg) was better than Indo (10.0 mg/kg).

### Effects of Inotilone on Carr-induced MMP-9 and NF-κB Protein Expressions in Mouse Paw Edema

The results showed that the injection of inotilone (5 mg/kg) on Carr-induced for 5 h inhibited MMP-9 and NF-κB proteins expression in mouse paw edema (Fig. 4B and 4C). The intensity of protein bands was analyzed by using Kodak Quantity software in three independent experiments and the result of it showed an average of 69.3% reduction of MMP-9 protein after the treatment with inotilone at 5 mg/kg compared with the Carr-induced alone. In addition, the protein expression showed an average of 57.5% reduction of MMP-9 protein after the treatment with Indo at 10.0 mg/kg compared with the Carr-induced alone (Fig. 4B). And the intensity of protein bands showed an average of 96.8% increase of NF-κB protein (*p*<0.001) (Fig. 4C).

### Inotilone Modulates the Activation of MAPK Pathways in Mouse Paw Edema

The activation of MAPK pathways in particular the phosphorylation of ERK1/2, JNK, and p38 expression were investigated by Western blot in paw edema tissues homogenates at the 5^th^ h after Carr injection. A significant increase in p-ERK1/2, p-JNK, and p-p38 levels was observed in Carr-treated mice (Fig. 4D). The treatment of mice with inotilone significantly reduced the level of p-ERK1/2, p-JNK, and p-p38 levels in mouse paw edema. On the contrary, inotilone treatment prevented the Carr-induced expression of these kinases. The intensity of protein bands were analyzed by using Kodak Quantity software in three independent experiments and showed an average of 59.8%, 61.7%, and 71.8% reduction of p-ERK1/2, p-JNK, and p-p38 proteins after the treatment with inotilone at 5 mg/kg compared with the Carr-induced alone (*p*<0.001) (Fig. 4D).

### Histological Examination

**Figure 5:**
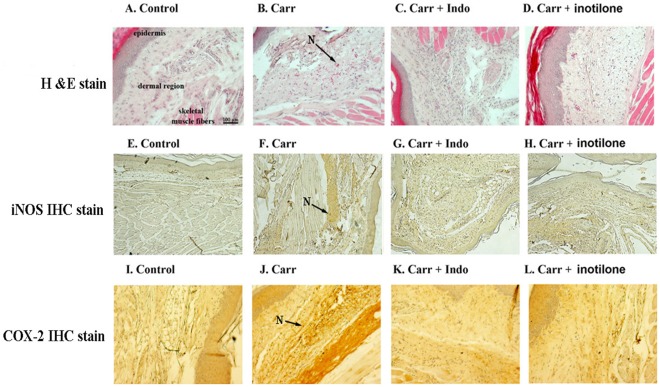
Histological appearances of mouse hind footpads after subcutaneously injecting 0.9% saline (Control group) or Carr, and then stained with H&E stain, while others were processed for iNOS and COX-2 immunohistochmistry staining. (A). Control mice: show the normal appearance of dermis and subdermis without any significant lesions, (F) iNOS and (J) COX-2 immunoreactive cells existed in the paws of normal mice; (B). Carr Only: Hemorrhage with moderately extravascular red blood cell and large amounts of inflammatory leucocytes, mainly neutrophils infiltrating the subdermis interstitial tissue. Moreover, the detail of the subdermis layer show enlargement of the interstitial space caused by the exudate fluid in the edema, (G) numerous iNOS and (K) COX-2 immunoreactive cells were observed in the brown site of paw tissue; (C). Carr + Indo 10 mg/kg (*i.p.*) (100×): there were obvious morphological alterations and improvements, (H) iNOS and (L) COX-2 immunoreactive cells; (D). Carr + inotilone: there were significant morphological alterations compared to the tissue with Carr treatment only. The lesions showed no hemorrhage and the number of neutrophils infiltrating the subdermis interstitial tissue was markedly reduced and also in (I) iNOS and (M) COX-2 immunoreactive cells in paws. Scale bar = 100 µm. There were markedly fewer inflammatory cells, and iNOS and COX-2 immunoreactive cells in the paws of Carr treated mice. The infiltrating cells were predominantly neutrophils (N; arrows). The brown staining indicated the interaction of primary and secondary antibodies and the presence of iNOS and COX-2.

Paw biopsies of the control mice showed marked cellular infiltration in the connective tissue. The infiltrates accumulated in collagen fibers and intercellular spaces. Paw biopsies of mice treated with inotilone (5 mg/kg) showed a reduction in inflammatory responses induced by Carr. Histologically, inflammatory cells were reduced in number and confined to the surroundings of the vascular areas. Intercellular spaces did not show any cellular infiltrations. Collagen fibers were regular in shape and showed a reduction in intercellular spaces. Moreover, the hypodermis connective tissues were not damaged (Fig. 5A). Neutrophils were increased with Carr treatment (Fig. 5B). Indo and inotilone (5 mg/kg) could decrease the neutrophils numbers as compared to the Carr-treated group (Fig. 5C and 5D). No inflammation, tissue destruction, iNOS and COX-2 immunoreactive cells (Fig. 5E and 5I). At the 5^th^ h after intraplantar Carr injection, numerous iNOS and COX-2 immunoreactive cells were observed in the brown site of paw tissue (Fig. 5F and 5J). Administration of Indo and inotilone (5 mg/kg) 30 min prior to the Carr injection markedly reduced the increase in iNOS amd COX-2 immunoreactive cells in paws (Fig. 5G, 5H, 5K, and 5L).

**Figure 6:**
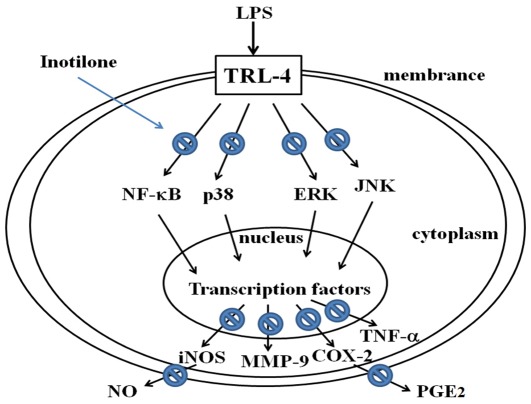
Proposed mechanism of inotilone inhibition of LPS-induced inflammation in RAW 264.7 cells. Inotilone abrogates the phosphorylation of MAPKs/IKK and subsequently inactivates NF-κB, which may result from inotilone down-regulation of iNOS and COX-2. Arrows indicate the main inflammatory pathway activated by LPS stimulation. The prohibition signs indicate the inhibitory effects of inotilone. TLR4; Toll-like receptor 4.

## Discussion

Inflammation represents a highly coordinated set of events that allow tissues to respond to injury, and it requires the participation of various cell types expressing and reacting to diverse mediators in a sequential manner [23]. In the present study, we demonstrated the anti-inflammatory activities of inotilone in both *in vitro* and *in vivo* experimental systems, by using LPS-stimulated RAW264.7 macrophages and a mouse model of topical inflammation respectively. The inhibitory activities against iNOS as shown in *in vitro* assays appear to confer on inotilone a potent *in vivo* efficacy in mouse suggesting its potential therapeutic usage as a novel topical anti-inflammatory source of health food. The pathology of inflammation is initiated by complex processes triggered by microbial pathogens such as LPS which is a prototypical endotoxin. LPS can directly activate macrophages which trigger the production of inflammatory mediators, such as NO, prostaglandin E2 (PGE_2_), TNF-α and leukotrienes [24]. However, no report has been issued on the anti-inflammatory effect of inotilone *in vivo* and the mode of action involved. Thus, this study was aimed to evaluate the anti-inflammatory effect of inotilone by screening the effects of inotilone on LPS-induced pro-inflammatory molecules *in vitro* and on acute phase inflammation *in vivo*. And, we also evaluated the mechanism of inotilone on MMP-9 and NF-κB expressions associated MAPK signaling pathways in the anti-inflammation.

LPS-induced macrophage activation increased the production of pro-inflammatory cytokines, NO by iNOS and PGE_2_ by COX-2, which are the main cytotoxic and pro-apoptotic mechanisms participating in the innate response in many mammals [25]. Therefore, LPS which stimulated macrophages can be effectively used as a model to study inflammation and potential anti-inflammatory mediators with their action mechanisms. Although iNOS plays a pivotal role in immunity against infectious agents by producing an excess amount of NO, this enzyme has come into the spotlight for its detrimental roles in inflammation-related diseases [26]. *In vitro* models such as macrophage cells or other cell lines are useful materials with a steady high-level production of NO. The mechanisms by which inotilone inhibits macrophage functions have not been elucidated. Examination of the cytotoxicity of inotilone in RAW264.7 macrophages using MTT assay has indicated that inotilone even at 25 µM did not affect the viability of RAW264.7 cells. Inotilone inhibited iNOS expression in LPS-stimulated macrophages and subsequently inhibited the NO production, whereas it decreased the enzyme activity of COX-2 instead of its expression to reduce PGE_2_ production [12]. In addition, Carr-induced inflammatory response has been linked to neutrophil infiltration release NO as well as that of PGE_2_. Results *in vitro* showed that inotilone suppressed LPS-induced production of NO, and the protein expression of iNOS and COX-2. The similar results for inotilone inhibits LPS-induced NO and PGE_2_ production through modulating iNOS expression and COX-2 enzyme activity [12].

Inhibiting NF-κB and MAPK pathways have been suggested as the two major mechanisms underlying the attenuation of LPS-induced inammatory cytokine production. NF-κB plays a crucial role as the transcription factor in regulating many of the pro-inammatory cytokine genes. LPS stimulation elicits a cascade leading to the activation of NF-κB [7]. The MAPKs play a critical role in the regulation of cell growth and differentiation and in the control of cellular responses to cytokines and stressors. Moreover, MAPKs are involved in the LPS-induced signalling pathway by which iNOS is expressed [27]. In the present study, we have demonstrated that the phosphorylation of MAPKs can be induced by LPS. The treatment with inotilone was found to significantly inhibit LPS-induced JNK, ERK, and p38 phosphorylation at 5, 10, 15, 30, and 60 min. Therefore, this suggests that JNK, ERK, and p38 are involved in the inhibition by inotilone of LPS-stimulated NF-κB binding in RAW 264.7 cells. Ajizian et al. (1999) suggested that activation of ERK is thought to be involved in LPS-induced macrophage responses [28]; in addition, JNK and p38 are activated by LPS stimulation and they have been postulated to play important roles in controlling iNOS gene expression [29]. In this study, we found that the treatment of inotilone blocked the activation of ERK1/2, JNK, and p38 MAPK, suggesting that inotilone suppresses LPS-induced NF-κB translocation by inhibiting the activation of these intracellular signaling cascades and it decreases the protein level of iNOS.

MMPs are involved in several pathological processes including cancers and inflammation. Among the MMPs, MMP-9 is secreted by macrophages regulates leukocyte migration in inflammatory diseases [5]. MMP-9 regulation involves transcriptional regulation, post-translational cleavage, and antagonism by physiological inhibitors [30]. In transcriptional regulation, MMP-9 expression is controlled by transcriptional factors including activator protein-1 (AP-1) and NF-κB, which bind to the corresponding binding sites in the MMP-9 promoter region [31]. In various kinds of cells, different stimuli induce MMP-9 expression through activation of the MEK-ERK or phosphoinositide 3-kinase (PI3K)-Akt signaling pathways, which subsequently activate AP-1 and NF-κB [32]. Also, p38 MAPK up-regulates MMP-9 expression in Raw 264.7 cells stimulated with LPS [33]. However, the upstream regulatory pathways that control the expression and secretion of MMP-9 are very complex and not well understood. Inotilone also decreased the phosphorylation of Akt and PI3K expression [12]. Our results are that inotilone inhibited the activities and the expressions of MMP-9 through decreasing of ERK signaling pathway which subsequently decrease NF-κB expression.

Carr-induced paw edema is a well-established model of edema formation which is commonly used for the screening of anti-inflammatory drugs. The intraplantar injection of Carr-induces inflammatory responses, including increases in paw volume and neutrophil infiltration [34]. Recent studies have shown that Carr-induced peripheral release of NO as well as that of PGE_2_ [26]. NO plays a major role in edema formation in inflammatory responses and tissue injury and Carr-induced the release of TNF-α level in the tissue [24]. Our results revealed that inotilone and Indo significantly inhibited the development of edema the 4^th^ h and the 5^th^ h after treatment. It was found that the injection of Carr into the mice paw induces the liberation of bradykinin, which later induces the biosynthesis of prostaglandin and other autacoids, which are responsible for the formation of the inflammatory exudates [21]. Our Carr-induced mice paw edema model enabled us to demonstrate the ability of inotilone to inhibit edema induced by acute inflammation. These results in conjunction with the marked inhibition of LPS-induced NO and TNF-α productions by inotilone in macrophages imply that the anti-edema effects of inotilone might result from its inhibition of NO and TNF-α syntheses in the peripheral tissues. The proinflammatory cytokines such as TNF-α and IL-1β are small secreted proteins, which mediate and regulate inflammation. TNF-α induces a number of physiological effects including septic shock, inflammation, and cytotoxicity [14]. Also, TNF-α is a mediator of Carr-induced inflammatory incapacitation, and it is able to induce the further release of kinins and leukotrienes, which is suggested to have an important role in the maintenance of long-lasting nociceptive response [3]. In this study, we found that inotilone decreased the TNF-α level after the Carr injection.

The Carr-induced inflammatory response has been linked to neutrophils infiltration and the production of neutrophils-derived free radicals, as well as the release of other neutrophils-derived mediators [4]. Many researchers demonstrated that inflammatory effect induced by Carr is associated with free radical. Free radical, prostaglandin and NO will be released when administrating with Carr for 1–6 h [3]. The reaction of NO with superoxide anion forms peroxynitrite, a potent cytotoxic oxidant eliciting lipid peroxidation and cellular damage. MDA, an indicator of lipid peroxidation, and antioxidant enzymes (CAT, SOD, and GPx) were also measured for evaluating the ability to scavenge radicals. Thus, inflammatory effect would result in the accumulation of MDA [35]. In this study, there were significantly decreases in MDA level with inotilone treatment. Furthermore, there was significantly increase in CAT, SOD, and GPx activities with inotilone treatment. We assume the suppression of MDA production is probably due to the increases of CAT, SOD, and GPx activities.

The MAPk family plays important roles in regulation of cell proliferation and cell death in response to various cellular stresses. During Carr-treated mice, oxidative stress and inflammatory cytokines activated MAP kinase kinases, leading to phosphorylation of ERK1/2, JNK, and p38 [36]. In the present study, we have observed an increase of phosphorylated MAPKs in the paw edema tissues at the 5^th^ h after Carr which is significantly reduced by the treatment with inotilone. Therefore, inotilone might alter NADPH oxidase activity through the inhibition of MAPK phosphorylation. Recent study also showed that the mechanism of action of inflammation involved the inhibition of the NADPH-oxidase-dependent superoxide production, the reduction of the intracellular GSH/GSSG ratio and prevention of the activation of the nuclear transcription factor NF-κB, which is an important mediator of inflammation [37].

Natural products are a valuable source of novel bioactive secondary metabolites. Various bioassays exist in which the anti-inflammatory activity of these products can be evaluated, having demonstrated that inotilone possesses anti-inflammatory activity *in vitro* and *in vivo* model of inflammation. In this model of *in vivo* acute inflammation, TNF-α and NO release in mice serum dropped markedly upon pretreatment with inotilone. It has been demonstrated that several natural product compounds which fall under the class of phenolic compounds act as strong inhibitors of NF-κB activation [38]. Inotilone also inhibited MMP-9 induction *via* suppression of NF-κB activity and MAPKs phosphorylation. LPS has been reported to up-regulate MMP-9 production in macrophages and neutrophils, astrocytes, and mast cells indicating the possible involvement of this enzyme in mediating the local infiltration of these inflammatory cells. From the present results, it was indicated that inotilone may regulate the above mentioned inflammatory responses through both inactivation of NF-κB and MAPKs.

In conclusion, inotilone suppresses LPS-induced MMP-9 expression by inhibiting the activation of NF-κB via ERK, p38, and JNK signaling pathways in RAW 264.7 cells. This is the first study showing that inotilone inhibits LPS-stimulated RAW 264.7 cells through specific inhibition of NF-κB-dependent MMP-9 expression via ERK, p38, and JNK signaling pathways (Fig. 6). And it is associated with the increase in the activities of antioxidant enzymes (CAT, SOD, and GPx) and inhibit of iNOS, COX-2, MMP-9, NF-κB, and MAPK expressions *in vivo*. These results suggest that inotilone represents a potential anti-inflammatory agent and this new beneficial effect may expand future researches on anti-inflammatory properties of inotilone *in vitro* and *in vivo*.
